# The Treatment of Asthma

**Published:** 1885-12

**Authors:** Paul Rodet, F. R. Campbell


### Translations.

### The Treatment of Asthma, by Dr. Paul Rodet.

Translated from *L'Abeille Med.*, by F. R. Campbell, M. D.

I. Treatment of the Attack.—The principal indication is to relieve the dyspnœa. For this purpose, the following remedies may be employed, given in the order of their efficiency:

(a) *Injections of Morphine.*—These rapidly relieve the attack. and produce a quiet sleep, but it is necessary to gradually increase the dose. It should be used with great caution, for fear of inducing morphinism.

(b) *Inhalation of Iodide of Ethyl.*—Direct the patient to pour ten or twelve drops on a handkerchief and inhale slowly. This drug rapidly relieves an attack, sometimes instantaneously.

These inhalations are much to be preferred to those of ether or chloroform, which usually fail.

(c) *Ammoniacal Vapor.*—This produces a sedative effect by exciting an excessive secretion in the nose and throat. Many patients are relieved in this way.

It has also been proposed to touch the pharynx with a strong solution of ammonia. A certain amount of inflammation with an abundant secretion is thus produced. This method of treatment sometimes affords excellent results.

(d) *The Inhalation of Medicated Fumigations.*— These act upon the bronchial mucous membrane. Nitre papers burned in a saucer near the patient, are much employed. The leaves of acrid narcotic plants, such as stramonium, belladonna, may be smoked in cigarettes alone, or with a small quantity of nitre. Cigarettes made of belladonna leaves, containing arsenic, are also prescribed. These means are beneficial only for a limited time.

2. Treatment of the Disease.—Seek out the causes which produce the attack, with a view to changing the occupation or surroundings of the patient, if necessary. The medical treatment will vary, according to the variety of asthma.

(a) *Catarrhal Asthma.*—Avoid cold; treat the laryngitis and bronchitis by ordinary methods, emollient drinks, ipecac and opium, and cutaneous revulsion. If the asthma is not of long standing, Hardy recommends the application, on the chest and arms, of a vesicatory or rubefacient. Tincture of lobelia, thirty to sixty drops a day, is considered an excellent remedy by the Germans.

The waters of Royat or Cauterets and a winter residence in the south, at the seaside, may be tried.

(b) *Nervous Asthma.*—Bromide and, above all, iodide of potassium produce excellent effects, although we do not know the rationale of this treatment.

Unroasted coffee, a tablespoonful to be infused in a cup of water over night, and taken at one dose in the morning for several months, may be tried.

Compressed air is an excellent remedy. The same results may sometimes be obtained by playing the cornet or blowing a trumpet, thus producing a distension of the bronchia. Gymnastic exercise of the upper extremities should be ordered for those of sedentary habits. Hydropathic treatment may be employed with patients who do not cough.

(c) *Herpetic Asthma.*—That is, cases in which the asthma alternates with attacks of skin disease. Employ the hygienic and therapeutic remedies mentioned above, and, in addition to these, arsenic. Counter-irritants, where the eczema has disappeared. Mont Dore water to be preferred to those of Bourboule, which are better for the scrofulous.

